# Emerging Biocontrol Strategies in Cheese Safety: A Critical Review of Bacteriophages, Antimicrobial Peptides, and Postbiotics

**DOI:** 10.3390/foods15193435

**Published:** 2026-09-25

**Authors:** Chiara Pisana, Arkadiusz Józef Zakrzewski, Margherita Caccamo, Wioleta Chajȩcka-Wierzchowska, Patryk Adamski, Cinzia Caggia

**Affiliations:** 1 Department of Agriculture, Food and Environment, University of Catania, Via S. Sofia, 100, 95123 Catania, Italy; chiara.pisana@phd.unict.it (C.P.); cinzia.caggia@unict.it (C.C.); 2 Department of Food Microbiology, Meat Technology and Chemistry, Faculty of Food Science, University of Warmia and Mazury, Plac Cieszyński 1, 10-726 Olsztyn, Poland; arkadiusz.zakrzewski@uwm.edu.pl (A.J.Z.); wioleta.chajecka@uwm.edu.pl (W.C.-W.); patryk.adamski@uwm.edu.pl (P.A.); 3 Consorzio per la Ricerca nel Settore della Filiera Lattiero-Casearia e dell’Agroalimentare (CoRFiLaC), 97100 Ragusa, Italy

**Keywords:** cheese safety, biopreservation, bacteriophages, antimicrobial peptides, postbiotics, foodborne pathogens, validation, HACCP

## Abstract

Fresh and soft cheeses feature high moisture content, elevated water activity, and nutrient richness, creating favorable conditions for foodborne pathogens including *Listeria monocytogenes*, *Salmonella* spp., Shiga toxin-producing *Escherichia coli*, and *Staphylococcus aureus*, which can contaminate products via post-pasteurization biofilm formation on processing equipment. Because conventional thermal and chemical preservation methods can compromise organoleptic and nutritional quality, biological control strategies have emerged as promising alternatives to enhance microbiological safety. This review critically evaluates three biopreservation modalities along their technological maturity gradient: bacteriophages and endolysins, antimicrobial peptides (AMPs) and bacteriocins, and postbiotics. Bacteriophages present high operational readiness, supported by regulatorily cleared commercial preparations; however, their lytic activity is frequently constrained by low temperatures, acidic pH, and matrix-bound fat. AMPs and bacteriocins—anchored by nisin—are extensively researched, with contemporary innovation focusing on advanced delivery vehicles, including active packaging, nanoencapsulation, and in situ bacteriocinogenic starter cultures, to preserve peptide stability throughout ripening. Postbiotics—here referring specifically to preparations consistent with the International Scientific Association for Probiotics and Prebiotics (ISAPP) consensus definition of inanimate microorganisms and/or their components, as distinct from the cell-free supernatants and fermentates most often tested in cheese—offer good physicochemical stability and dose control, albeit with lower and more variable intrinsic antimicrobial potency. Across all modalities, multi-target hurdle approaches consistently outperform single-agent applications, with surface-targeted delivery representing the dominant engineering strategy. Persistent limitations across all strategies include post-treatment bacterial regrowth, matrix interference, non-standardized activity units, and unresolved regulatory status. Moving forward, standardized, full-shelf-life challenge trials in real cheese matrices under harmonized storage conditions are imperative to benchmark these biointerventions head-to-head against conventional methods and live protective cultures.

## 1. Introduction

Cheese safety is a critical concern because contamination with undesirable microorganisms can occur throughout production, storage, and distribution. Fresh cheeses in particular are gaining appreciation among consumers for their quality traits [1,2], yet their large-scale marketing is strongly compromised by microbiological issues and a limited shelf life under current vacuum-packaging systems. Their high moisture content and rich nutritional profile provide ideal conditions for the growth of spoilage and pathogenic bacteria, limiting preservation even under refrigeration [3]. The capacity of pathogens to proliferate along the food chain is closely linked to their ability to grow at refrigeration temperatures and to withstand acidic and osmotic stress [4], while their capacity to form biofilms on food-contact surfaces and equipment makes them a persistent challenge for production facilities, driving post-processing recontamination even after effective heat treatment [4,5]. Human infection occurs primarily through the consumption of contaminated foods, including raw milk and dairy products [6,7]. In medium-aged cheeses, the rind can further act as a substrate for microbial growth [5,8]. Consequently, extending the shelf life of fresh cheeses while safeguarding microbiological safety represents one of the most important challenges facing the sector [9], notwithstanding the preservation techniques and regulations that have substantially improved overall dairy safety [10,11]. It should be noted that dairy products may also carry chemical hazards, such as drug residues, mycotoxins, and environmental pollutants that originate in raw milk and persist through processing because pasteurization, fermentation, and aging target microorganisms rather than chemical residues [12,13]. These hazards, however, fall outside the scope of the present review, which focuses on biological interventions against microbiological hazards. As consumers place increasing emphasis on food safety and on the link between diet and health [14], manufacturers must prioritize ingredient selection, processing, and packaging integrity [15,16]. However, conventional decontamination methods frequently degrade product quality [17], adversely affecting sensory characteristics, nutritional value, and environmental sustainability—key drivers of consumer choice, especially for dairy products [18]. Thermal and chemical treatments therefore impose a trade-off between microbiological safety and the organoleptic and nutritional attributes that define these products, motivating the search for milder alternatives. To overcome these limitations, biological control strategies that exploit natural agents—bacteriophages, antimicrobial peptides and bacteriocins, and postbiotics—are gaining traction as means of improving the microbiological profile of cheese while preserving its quality. This review critically examines these emerging strategies and their real-world applicability. Crucially, it outlines the validation steps—particularly regarding sensory effects and full-scale production testing—required before such methods can be implemented as preventive measures within hazard analysis and critical control point (HACCP) plans.

A structured literature search was conducted in PubMed, Scopus, and Web of Science (Core Collection) to identify studies on biocontrol strategies against foodborne pathogens in cheese and dairy matrices. The search covered publications from database inception to August 2026 and was last updated on 1 September 2026. Boolean search strings combined matrix, intervention, and target-pathogen concepts, for example: (“cheese” OR “dairy” OR “milk”) AND (“bacteriophage” OR “phage” OR “endolysin” OR “antimicrobial peptide” OR “bacteriocin” OR “nisin” OR “postbiotic” OR “cell-free supernatant” OR “fermentate”) AND (“*Listeria monocytogenes*” OR “*Salmonella*” OR “*Escherichia coli*” OR “STEC” OR “*Staphylococcus aureus*”) AND (“biocontrol” OR “biopreservation” OR “antimicrobial” OR “anti-biofilm” OR “challenge test” OR “validation”). Terms were adapted to the syntax and field tags of each database and searched in titles, abstracts, and keywords. Inclusion criteria were: peer-reviewed original research or reviews published in English that investigated the antimicrobial and/or anti-biofilm efficacy of bacteriophages/endolysins, antimicrobial peptides/bacteriocins, or postbiotics against the selected pathogens in cheese, milk, or other dairy-relevant food matrices and experimental models. Studies were excluded when they addressed non-dairy food matrices, clinical or veterinary applications without direct relevance to food systems, or spoilage microorganisms in the absence of a specific food-safety perspective. After removal of duplicates, records were screened by title and abstract for topical relevance, and the full texts of the retained records were then assessed against the inclusion and exclusion criteria. Reference lists of pertinent reviews were hand-searched to capture additional primary studies. Records meeting all criteria were included in the qualitative synthesis presented in this review.

## 2. Microbiological Hazards

The safety of dairy products remains a paramount concern for public-health authorities [10,19], because fresh cheeses—especially soft-textured and raw-milk varieties, with their high moisture, high water activity, and abundant nutrients—support a wide range of contaminants [4,20]. Rather than reviewing the general biology of these agents, which has been detailed elsewhere, we focus here on the traits that condition biological intervention in cheese: tolerance of low pH and low temperature, survival in fat- and casein-rich matrices, and biofilm formation on processing surfaces. Four foodborne pathogens dominate the dairy-borne outbreak record and current European surveillance—*L. monocytogenes*, *Salmonella* spp., Shiga toxin–producing *E. coli* (STEC), and *S. aureus* [10,21,22]. The EFSA/ECDC One Health 2024 Zoonoses Report ranks salmonellosis and STEC among the most frequently reported zoonoses and invasive listeriosis as the most severe (highest hospitalization and case-fatality), with all four agents documented in raw-milk and soft, semi-soft and hard cheeses under Regulation (EC) 2073/2005 monitoring [22]. For biological control, the decisive feature of *L. monocytogenes*—the primary cause of human listeriosis, with high case-fatality [23,24] and serotypes 1/2a, 1/2b, 1/2c and 4b most relevant to foods and outbreaks [25,26]—is its psychrotrophic growth (from about −0.4 °C, pH 4.3–9.6, low water activity, high salt) combined with biofilm-mediated persistence on equipment, which drives post-pasteurization recontamination of ripened cheese and rind [27,28,29]; any phage, peptide or postbiotic must therefore stay active through cold, extended ripening and act at the surfaces where recontamination originates. *Salmonella* spp., consistently among the most frequently reported zoonotic agents [30,31] and, with listeriosis, among the deadliest foodborne infections [32], are generally pasteurization-sensitive but persist in low-moisture, high-fat foods [33] and mount a robust acid-tolerance response, so acid-adapted cells survive the mildly acidic, high-fat conditions of many cheeses [32]. Because the infectious dose is lowered by fatty matrices [34] and contamination is largely post-processing [31], interventions relying on acidification alone are insufficient and surface-targeted or hurdle approaches are required. Pathogenic *E. coli*, notably the Shiga toxin–producing serotypes (VTEC/STEC, e.g., O157:H7) that cause hemorrhagic colitis and hemolytic uremic syndrome [35,36,37], are shed by ruminants and reach cheese via raw milk [38]. From an intervention standpoint, the key constraints are that STEC are Gram-negative—intrinsically less susceptible to most bacteriocins unless combined with outer-membrane permeabilizers—and that a very low infectious dose leaves little margin for survivors, placing a premium on lytic phages and hurdle combinations in raw-milk cheeses [39]. Finally, *S. aureus* is a Gram-positive, catalase-positive, highly halotolerant coccus (growth up to ~15% NaCl) that persists in cheese brines [40,41] and whose skin/mucosal and mastitic reservoirs make handlers and livestock primary contamination sources [40,42]; its critical hazard is the production in heat-stable staphylococcal enterotoxins that resist pasteurization and remain active after cells are killed [43,44], so once densities exceed ~10^5^ CFU/g enterotoxin formation is likely [40,42,45,46]. The salient implication is that an intervention must suppress *S. aureus* growth below this toxigenic threshold rather than merely inactivating cells after the fact.

### Commission Regulation (EC) 2073/2005

Commission Regulation (EC) 2073/2005 defines food safety and process hygiene criteria for key dairy pathogens. For ready-to-eat (RTE) foods that do not support *L. monocytogenes* growth, the limit remains 100 CFU/g throughout shelf life. However, for growth-supporting RTE foods like traditional fresh cheeses, Commission Regulation (EU) 2024/2895 (effective 1 July 2026) tightened category 1.2 of Annex I. Operators can now maintain the 100 CFU/g limit only if they prove to competent authorities—using challenge testing or validated predictive modeling—that the threshold will not be breached. Without such proof, a strict “not detected in 25 g” criterion applies to products on the market across their entire shelf life, expanding this mandate from the manufacturing phase to the full distribution chain [23,47]. The same regulation sets a zero-tolerance criterion for *Salmonella* spp. in dairy products, requiring their absence in 25 g and necessitating stringent primary hygiene measures together with innovative biocontrol strategies (e.g., bacteriophages, natural antimicrobials, improved sanitation) to maintain compliance and protect public health [48]. Within the European Union, *E. coli* is regulated primarily as a process hygiene indicator rather than via strict finished-product limits: Regulation (EC) 2073/2005 establishes *E. coli* criteria to assess fecal contamination and process control, while risk-based management of STEC relies on a comprehensive “farm to fork” preventive strategy, endorsed by scientific expert bodies as the most effective way to reduce STEC outbreaks in the dairy sector [38]. For *S. aureus*, EU microbiological criteria in dairy products are codified in Commission Regulation (EC) 2073/2005 and its amendment 1441/2007, which establish staphylococcal enterotoxins as a food safety criterion in cheeses and other dairy products, requiring their absence in the finished product [40]. In cheeses made from raw milk, counts of coagulase-positive staphylococci ≥10^5^ CFU/g trigger mandatory testing of the batch for enterotoxins using validated immunological methods, reflecting the bacterial density threshold associated with toxin formation [46]. This tiered approach underscores the importance of controlling initial *S. aureus* loads and preventing growth during cheesemaking and storage, in line with a “farm-to-fork” risk management concept that integrates udder health, milking hygiene, process control, and temperature management to avoid irreversible toxin accumulation in the final product [45].

## 3. Bacterial Biofilms in Cheese Processing Environments

A critical factor contributing to the recurring contamination of traditional fresh cheeses is the capacity of foodborne pathogens to shift from a planktonic state to a sessile lifestyle, forming biofilms on food-contact surfaces [49,50]. A biofilm is a structured microbial community attached to a surface and embedded in a self-produced matrix of extracellular polymeric substances (EPS), mainly polysaccharides, proteins, lipids, and extracellular DNA (eDNA), which together form a highly hydrated, cohesive network. Biofilm development is a dynamic, multistage process starting with reversible attachment to materials such as stainless steel, plastic, or traditional wooden vats, followed by irreversible adhesion, EPS matrix maturation, and ultimately cell dispersal that seeds new sites within the dairy plant [51,52]; in dairies, residual milk proteins and fats condition surfaces and provide nutrients that markedly accelerate biofilm formation and colonization [53]. The EPS matrix acts as a powerful physical and chemical barrier, restricting the penetration and efficacy of conventional sanitizers and contributing to increased resistance to disinfectants, pH shifts, desiccation, osmotic stress, and temperature fluctuations typical of cheese-making environments [54,55]. Within this protected milieu, the proximity of cells favors horizontal gene transfer, including the spread of virulence and antimicrobial resistance determinants [52]. In traditional Sicilian dairy systems, biofilms on wooden equipment (tine) represent a technological double-edged sword. Beneficial biofilms dominated by lactic acid bacteria (LAB) on vats, shelves and tools contribute to fermentation, inhibit pathogens, and preserve product typicity and sensory authenticity [50,51,56,57]. However, the coexistence or occasional inclusion of pathogens such as *L. monocytogenes*, *S. aureus*, or spoilage organisms within these communities constitutes a major safety concern, as these bacteria can persist for years in “harborage sites” like drains, valves, and joints, often surviving standard cleaning-in-place (CIP) regimes [58,59]. Their persistence and periodic release from environmental reservoirs into milk or curd underscore the limitations of traditional hygiene protocols and the need for targeted biotechnological strategies. Innovative approaches under investigation include EPS-targeted interventions, such as enzyme-based CIP formulations (e.g., proteases, polysaccharidases, DNases) and bacteriophage- or phage-derived enzymes, which are capable of degrading the EPS matrix and enhancing biofilm penetration, thereby facilitating the removal of embedded pathogens while preserving or reestablishing desirable LAB biofilms on wooden tools [52,53,60].

## 4. Bacteriophages

Bacteriophages are the most operationally mature among the biological control strategies for cheese: several preparations are commercially available and regulatorily cleared, and lytic phages routinely deliver multi-log pathogen reductions. But phage effectiveness is also the one most shaped by a set of hard conditioning factors, because a phage is a self-replicating biological agent whose performance depends on host density, temperature, pH, and the physical structure of the matrix in ways a chemical preservative does not. Unlike AMPs and postbiotics, a key defining feature of this field is the availability of commercial products. Phage preparations such as Listex (PhageGuard P100), ListShield, EcoShield, and SalmoFresh have secured regulatory clearances (e.g., GRAS or equivalent) across several jurisdictions [61], and much of the existing literature focuses on their application to cheese and milk. While their efficacy is well established, its magnitude depends heavily on the food matrix and is often modest. For instance, the ListShield cocktail achieved an 82% (0.7-log) reduction in *L. monocytogenes* on cheese without altering organoleptic properties [62]. In contrast, within the more permissive matrix of pasteurized milk, a commercial preparation achieved approximately a 4-log reduction in *L. monocytogenes* by day 7 [61]. The gap between these two outcomes—despite involving the same pathogen and agent class—highlights the primary practical limitation of phage biocontrol in cheese. Addressing this disparity is the driving force behind research into hurdle technology and targeted delivery systems. Recent studies on commercial formulations have thus prioritized phage viability and spatial localization, such as embedding Listex P100 into biodegradable antimicrobial films [63] or applying it to coated paper packaging, where humidity management is essential for preserving titer stability [64].

### 4.1. In-Cheese Efficacy

Research into phage application in cheese has successfully elucidated the key mechanisms driving variable efficacy, linking them to specific intrinsic properties of the food matrix. Temperature and pH serve as the dominant levers: phage-mediated reduction in *L. monocytogenes* is significantly higher at 22 °C than at 6 °C or 14 °C, and at pH 6.0–6.5 compared to pH 5.5 [65]. This finding underscores a fundamental challenge in dairy safety: the low temperatures and acidic conditions used to retard pathogen growth simultaneously impair phage activity, compromising efficacy when protection is most critical. Additionally, the physical architecture of cheese poses physical barriers: fat and casein entrap phage particles, hindering spatial diffusion toward host cells, while matrix encapsulation provides a double-edged defense against environmental stresses [66]. One constructive exploitation of matrix physics is the high-pressure-processing hurdle: Listex P100 remained infective at pressures up to 300 MPa in a traditional cheese; therefore, a phage-plus-HPP combination can attack the pathogen through two independent mechanisms [66]. A formulation-level lever specific to the dairy matrix is calcium concentration, which modulates phage adsorption and efficacy against *S. aureus* in cheese [67].

### 4.2. Pathogen Spectrum and Cheese-Specific Applications

Although *L. monocytogenes* dominates the phage literature as it does the others, phages reach a notably wider set of dairy pathogens, and crucially they reach Gram-negatives that bacteriocins largely cannot. For Shiga-toxin and enterotoxigenic *E. coli*, the EcoShield cocktail cut *E. coli* O157:H7 in cheese by up to 97% at 5 × 10^6^–10^7^ PFU/g [68], and a newly isolated lytic phage achieved a 4.9–5.8 log CFU/mL reduction in enterotoxigenic *E. coli* in cheese at 4 °C [69], the largest in-cheese reductions in this corpus, and against a Gram-negative. For *Salmonella*, commercial preparations reduce counts in milk and raw-milk cheese, with the effect strain-dependent [61]. For *S. aureus*, a two-phage treatment eradicated 10^6^ CFU/g in cheddar curd after 14 days at 4 °C without enterotoxin overproduction [70], and a newly isolated myovirus, phiIPLA-LAVI, kills *S. aureus* in cheese matrices while remaining stable across pH 4–7 [71]. A distinctively dairy target is *Clostridium tyrobutyricum*, the cause of late-blowing defect in semi-hard cheeses: lytic phage FA67 delayed late-blowing symptoms by two weeks by reducing vegetative *Clostridium* at 14 days of ripening [72]. Phages also increasingly target biofilms and spoilage on food-contact surfaces rather than the cheese itself, as with phage-loaded alginate films that reduce *E. coli* and *Pseudomonas fluorescens* biofilms by 1.7 and 2.7 log units with substantial biomass removal [73].

### 4.3. From Phages to Endolysins

The most pronounced recent shift in phage research is the transition from whole phages to their purified lytic enzymes. Endolysins—the peptidoglycan hydrolases that phages deploy to lyse host cells—circumvent several key limitations of whole-phage therapies. They function independently of host-cell density, do not self-replicate, act rapidly, and avoid triggering receptor-loss resistance. Among dairy-specific enzymes, PlyP100 is the most thoroughly characterized endolysin, demonstrating sustained anti-listerial activity in fresh cheese for over four weeks under refrigeration while remaining optimal at native pH and salt levels [74,75]. Similarly targeting *Clostridium*, the LysFA67 endolysin achieved a 93% cell lysis rate within just four minutes [76]. The most sophisticated development is in situ delivery, engineering the cheese starter itself to secrete the lysin so that it is produced continuously in the matrix: engineered *L. lactis* delivering the CTP1L endolysin against *Clostridium* [77,78], and engineered *L. casei* secreting the Lysdb endolysin achieved a 10^5^-fold reduction in *S. aureus* in cheese, holding the pathogen below 10^4^ CFU/g through six weeks of ripening [78]. Endolysin engineering also enables detection and typing: GFP-fused CTP1L cell-binding domains label *Clostridium* vegetative cells and spores in late-blowing cheese with strain specificity [79], and isolated catalytic domains such as the CHAP domain of LysGH15 retain anti-staphylococcal activity in milk [80]. The engineered-lysin approach is also the field’s answer to the Gram-negative problem, since lysins fused to outer-membrane permeabilizers can reach the peptidoglycan of *Salmonella* and *E. coli* [81,82].

### 4.4. Phages and Starter Cultures

A unique hallmark of the phage modality is that the very biology harnessed to control pathogens also poses a chronic threat to production. Phages infecting *Lactococcus* and *Streptococcus* starter cultures remain the primary cause of delayed or failed cheese fermentations worldwide, forcing processing facilities to deploy biocontrol phages while strictly excluding wild contaminant strains simultaneously. Extensive surveys mapping starter-phage biodiversity in production facilities [83,84] underscore this operational risk, and this dual identity—phages as both biopreservation tools and fermentation hazards—is now a central theme in the literature [85]. In biocontrol applications, these practical tensions persist. Deploying phages against specific pathogens requires careful management of their coexistence with starters, complicating both quality assurance and regulatory approval.

### 4.5. Limitations

The limitations most specific to phage biocontrol are threefold. First, whole-phage treatments drive the emergence of receptor-loss mutants and possess inherently narrow, strain-specific target spectra. This motivates the shift toward phage cocktails, phage–bacteriocin combinations, and endolysins. Second, phage efficacy is uniquely sensitive to the physical structure of the cheese matrix, frequently collapsing under cold, acidic, and high-fat conditions, so in vitro broth and milk assays systematically overstate real-world performance. Third, and distinctive to this modality, phage preparations face specific conceptual and perception barriers: they occupy an ambiguous status between processing aids and food additives across jurisdictions [86], they raise unresolved consumer concerns about the deliberate addition of “viruses” to food, and genetically modified starter strains engineered to secrete lysins attract additional regulatory scrutiny. Meta-analyses aggregating hundreds of phage–food studies [87] and recent systematic reviews [88,89] converge on the recommendation that future work prioritize cocktails, hurdle strategies, and endolysin systems evaluated against resistant regrowth and matrix-induced activity loss. The cross-cutting challenges shared with the other modalities—post-treatment regrowth over the full ripening period, non-standardized reporting units, and the need for full-shelf-life challenge trials—are treated comparatively in Section 7.

The main bacteriophage- and endolysin-based interventions evaluated in dairy systems are summarized in Table 1, with emphasis on their microbial targets, application matrices, mechanisms of action, and principal outcomes.

## 5. Antimicrobial Peptides (AMPs)

AMPs and bacteriocins represent the most thoroughly investigated biological control strategies in dairy processing, with research reflecting a clear shift in focus. While the discovery and characterization of these ribosomally synthesized peptides are largely established, recent innovation centers are almost exclusively on delivery systems—specifically, strategies to maintain peptide activity, spatial localization, and inhibitory concentrations throughout weeks of ripening within a complex, cold matrix rich in fat and proteins. The practical toolbox remains focused and well-characterized. Nisin—a lantibiotic and the only bacteriocin with GRAS status and an assigned food-additive number—serves as the benchmark compound. Consequently, a substantial portion of the cheese literature focuses either on evaluating nisin in novel delivery formats or on benchmarking newly identified bacteriocins against it. Surrounding nisin is a secondary tier of class II bacteriocins with established track records in dairy, including enterocins from *Enterococcus faecium* and *Enterococcus faecalis*, pediocin-family peptides, lacticin 3147, plantaricins, and lactococcins, alongside an expanding array of milk- and cheese-derived AMPs identified through activity screening. Two key features consistently recur across this toolbox, underpinning its suitability for dairy applications. First, these peptides display a strong bias toward Gram-positive targets—primarily *L. monocytogenes*, which remains the dominant pathogen evaluated across the literature—while the outer membrane of Gram-negative bacteria confers intrinsic resistance that can only be overcome through combination strategies. Second, they exhibit exceptional stability: novel enterocins, isolated from artisanal cheeses, consistently demonstrate thermal tolerance between 80 °C and 100 °C, alongside activity across a broad pH range [90]. This robust profile provides the resilience necessary to survive pasteurization-adjacent processing and extended cold storage. Newer studies are focused on resolving the mechanism at the molecular level: the milk-derived peptide GMp7 was shown to disrupt *S. aureus* through simultaneous interference with peptidoglycan synthesis, ATP transport, and DNA replication [91], the kind of multi-target action that makes rapid resistance less likely.

### 5.1. Bacteriocinogenic Starters and Adjuncts

The most extensive body of research on dairy biocontrol centers focuses on delivering these peptides via live producer strains integrated directly into the fermentation process—either as bacteriocinogenic starter cultures or as protective adjuncts capable of secreting peptides in situ during ripening. This strategy is particularly appealing because it enables continuous, localized peptide production at the site of potential contamination without requiring external additives, thereby supporting a clean-label claim. Traditional cheese environments serve as ideal discovery sources for these strains, with bacteriocinogenic *Enterococcus, Lactococcus*, and *Lactobacillus* isolates consistently recovered from raw-milk and artisanal cheeses [92,93]. Among these, *E. faecium* KE82—an enterocin A-, B-, and P-producing adjunct—is particularly well characterized. Tested in real cheese systems, it reduced *L. monocytogenes* by 0.54–2.00 log CFU during Galotyri ripening at 4–12 °C [94], while studies on mixed-starter compatibility confirmed the active transcription of its enterocin genes throughout milk fermentation [95,96]. Nisin-producing *L. lactis* starters provide effective, comparable control of *L. monocytogenes* and *S. aureus* in fresh cheese matrices [97]. Furthermore, in situ production in purified enterocins can reduce *Listeria* levels below detectable limits when expressed at sufficient titers (e.g., 2048 AU/g over 72 h) [92]. The frontier in this area lies in multi-bacteriocin delivery systems. To widen the spectrum of inhibition and minimize selection of single-drug-resistant mutants, engineered starters combining nisin, lacticin 3147, and a plantaricin have been developed [98]. In parallel, recombinant strains secreting enterocin A demonstrate multi-log reductions against *Listeria* [99]. Bacteriocinogenic strains also deliver in more mature cheeses: a *L. lactis* strain achieved a 3-log *Listeria* reduction over ten days in goat cheese [93].

### 5.2. Free-Peptide Application: Practical Ceilings

Direct application of a purified or semi-purified peptide to milk or cheese represents the most straightforward and extensively studied strategy, paving the way for the advanced delivery formats. For instance, free nisin effectively inhibits *L. monocytogenes* in Queso Fresco and processed cheese models, where bacterial growth can be predictably controlled using pH-dependent minimum inhibitory concentration (MIC) models at residual levels of ~0.5–5.0 ppm [100,101]. Similarly, exogenous application of staphylococcins to fresh Minas cheese reduces *S. aureus* populations by 95% to 99.99% [102]. However, the limitations of free peptides are both well documented and persistent: activity declines as the peptide binds to fat and casein and disperses throughout the matrix, while Gram-negative pathogens remain largely resistant. The key to overcoming this bottleneck lies not in higher dosages, but in synergistic combinations. For example, pairing free nisin with caprylic acid and trans-cinnamaldehyde completely suppressed *Salmonella* in queso fresco, whereas nisin alone failed [103]. This recurring pattern—a modest free-peptide effect salvaged by a co-agent or delivery system—represents a central paradigm shift in the field.

### 5.3. Encapsulation and Active Films

The two primary engineering strategies developed to overcome the free-peptide limitation are encapsulation—which protects the peptide and mediates sustained release—and active packaging, which concentrates the peptide at the surface to target post-processing contamination. Both approaches are now backed by strong evidence in dairy applications. Encapsulation systems include chitosan-coated nisin–silica liposomes that sustained anti-listerial activity in cheddar without compromising sensory traits [104], alginate/resistant-starch microcapsules providing controlled nisin release against *C. tyrobutyricum* at production scale [105], and Neusilin or zein-starch nanoparticle carriers engineered for extended release [106]. Active films and coatings have proven equally effective: a nisin–chitosan-cellulose film completely inactivated *L. monocytogenes* on ultrafiltered cheese, retaining antimicrobial activity at 4 °C for one month [107]; enterocin-loaded whey protein films reduced *Listeria* to undetectable levels within 20–30 days while physically preventing pathogen migration between slices [108]; spray-dried enterocins in an agar-based coating yielded a 1.0 log CFU/mL reduction in commercial goat cheese [109]; and *Latilactobacillus curvatus* bacteriocin films successfully controlled *Listeria* on sliced Prato cheese [110]. A notable high-performance variant is electrospun nanofiber delivery: nisin-loaded amaranth–pullulan fibers completely inactivated *L. monocytogenes* in fresh cheese within 120 h [111]. Moreover, whey protein-based films present a circular solution by repurposing a dairy by-product as the carrier matrix [112].

### 5.4. Synergistic Combinations and the Endolysin Partnership

Due to the narrow spectrum and resistance risks associated with single-peptide treatments, recent advances highlight the superior efficacy of combination strategies, most notably the synergy between bacteriocins and phage endolysins. The paradigm result of the whole cheese-biocontrol field is a bacteriocin combination: PlyP100 endolysin with nisin drove *L. monocytogenes* to non-enumerable levels in queso fresco over four weeks with no resistant escape [113], and an engineered bioactivity-improved nisin derivative (H27/31K) paired with the same endolysin reproduced non-enumerable control in fourteen days [114]. Beyond endolysins, bacteriocin plus essential oil is a recurring hurdle strategy: enterocin OS1 with eucalyptus essential oil reached undetectable *Listeria* in Moroccan fresh cheese within two days [115], as did bacteriocin combinations with organic acids and physical hurdles [116]. The underlying rationale across these approaches remains consistent: combining agents with complementary mechanisms broadens the antimicrobial spectrum, lowers individual dosage requirements, and prevents the emergence of resistance that frequently limits single-peptide therapies.

### 5.5. Secondary Functions of AMPs: Anti-Biofilm and Quality-Related Applications

Beyond their core role in inhibiting foodborne pathogens—the focus of the preceding subsections—AMPs and bacteriocins have been explored for a set of secondary, quality-oriented functions in cheese. These applications are conceptually distinct from planktonic pathogen killing and are grouped here to keep them separate from the mainstream antimicrobial-efficacy discussion. Although *L. monocytogenes* remains the primary research target, the AMP literature addresses a broader range of dairy challenges than either the phage or postbiotic fields. For staphylococci, both staphylococcins and lactic acid bacteria (LAB) bacteriocins effectively control drug-resistant *S. aureus* and MRSA strains isolated from cheese [102,117]. Furthermore, calcium concentration has been shown to modulate bacteriocin efficacy against *S. aureus* in cheese—representing a formulation lever unique to the dairy matrix [67]. A distinctive AMP application targets cheese quality rather than pathogen control: nisin A inhibited 31 of 48 biogenic-amine-producing LAB and prevented biofilm formation by two-thirds of biofilm ormers, addressing histamine and tyramine accumulation rather than infection [118]. Anti-biofilm activity against *Listeria* and staphylococci recurs as a mechanism distinct from planktonic killing [99], and novel bacteriocinogenic *Pediococcus* and *Enterococcus* strains continue to be characterized against *Listeria* with quantified titers [119].

### 5.6. Limitations

The limitations most specific to antimicrobial peptides center on resistance and producer-strain safety. *Listeria* readily develops nisin tolerance, and although multi-bacteriocin and bacteriocin–endolysin systems are designed to counter this, most single-peptide challenge studies are too brief to capture resistant regrowth [120]. From a regulatory standpoint, only nisin holds clear GRAS/additive status, whereas newer enterocins and engineered nisin derivatives remain unapproved. A concern unique to this modality is producer-strain safety: enterococcal producers in particular may harbor virulence determinants and antibiotic-resistance genes, which complicates their deployment as live adjunct cultures regardless of bacteriocin potency and are considered together with the other producer-strain safety issues in Section 7. The matrix effects and unit-standardization problems that AMPs share with phages and postbiotics are likewise addressed comparatively in Section 7.

Representative antimicrobial peptides and bacteriocins investigated for cheese biopreservation are presented in Table 2, which compares the agents, target microorganisms, delivery matrices, and reported antimicrobial effects.

## 6. Postbiotics

Among biological strategies for controlling pathogens in cheese, postbiotics represent the newest and least established category. Nevertheless, the field has progressed rapidly from initial definitional debates—addressed in detail in Section 6.1—to a growing body of direct in-product evidence. The antimicrobial activity of a dairy postbiotic is rarely attributable to one compound: it is the combined effect of several metabolite classes in the spent medium, and recent studies have become markedly better at attributing activity to specific molecules. Organic acids are the dominant contributor in most fresh-cheese systems: da Costa and co-workers [121] showed that fruit-derived *Lactiplantibacillus plantarum* and *Lacticaseibacillus paracasei* reduced *L. monocytogenes* in Frescal Minas cheese, with the effect tracking lactic and acetic acid production and the accompanying pH drop. Where organic acids account for most—but not all—of the antimicrobial effect, untargeted metabolomics are increasingly resolving the remaining active fraction. For example, a *Lactiplantibacillus plantarum* strain isolated from Iranian Koupa cheese owed its anti-*S. aureus* and anti-*E. coli* activity to nocardamine and diketopiperazines within its cell-free supernatant (CFS) [122]. Similarly, antifungal dairy fermentates have been resolved into propionic, acetic, lactic, and butyric acids acting synergistically with hydroxy fatty acids and phenolics; notably, a single hydroxylated fatty acid (10-OH-18:1) combined with acetate accounted for over half of the total antifungal activity in one comprehensive screen [123,124]. Farhat and colleagues [125] similarly correlated LAB antifungal activity with defined organic acids and phenolic metabolites at measured MICs. Beyond organic acids, the postbiotic fraction encompasses a diverse array of bioactive compounds, including bacteriocins, bacteriocin-like inhibitory substances (BLIS), exopolysaccharides, biosurfactants, hydrogen peroxide, and secreted peptides—a compositional inventory well established across the biopreservation literature [126,127]. A recurring practical virtue of these molecules is robustness: BLIS and bacteriocin-containing CFS from cheese isolates are repeatedly reported as heat-stable to 121 °C and active across a broad pH range [128,129], the stability that a non-viable preparation needs to survive cheese processing and storage. Empirical evidence from actual cheese and dairy matrices is examined in Section 6.2.

### 6.1. Defining Postbiotics and Related Formulations

The most pivotal recent milestone in this domain was conceptual rather than experimental: the 2021 ISAPP consensus, subsequently clarified in a widely cited follow-up publication, formally defined a postbiotic as a “preparation of inanimate microorganisms and/or their components that confers a health benefit on the host” [130,131]. Two features of that definition shape everything downstream. First, the agent is deliberately non-viable, which is the property that makes postbiotics attractive for cheese. Unlike live protective cultures, an inactivated preparation cannot proliferate, acidify unpredictably, or raise the safety concerns that live cultures carry for immunocompromised and other vulnerable consumers [126]. Second, the ISAPP consensus emphasizes whole preparations—inactivated cells alongside their spent fermentation medium—rather than isolated, purified molecules. Consequently, the standard functional unit in the cheese literature is typically a cell-free supernatant (CFS) or a fermentate rather than a chromatographically pure compound. This has led to persistent terminological ambiguity: many dairy studies classify single bacteriocins or organic acids as postbiotics, a practice that conflicts with the strict ISAPP framework despite its widespread operational use, and key reviews anchoring the application of postbiotics in cheese—such as those on natural cheese preservatives [132], LAB postbiotics in food biopreservation [127], and the broader management of *Listeria* in soft cheeses [120]—operate within this conceptual ambiguity rather than resolving it. Because the term “postbiotic” is used inconsistently throughout the dairy literature, and because several of the preparations discussed below are primarily investigated as antimicrobial food preservatives rather than as agents intended to confer a demonstrated health benefit to the host, it is important to distinguish four categories that are frequently conflated. (i) Postbiotics sensu stricto are preparations of inanimate microorganisms and/or their components that confer a health benefit to the host, consistent with the 2021 ISAPP consensus definition [130,131]. The health-benefit criterion is central to this definition and is rarely addressed when such preparations are evaluated solely for antimicrobial activity within a food matrix. (ii) Postbiotic-derived antimicrobial preparations comprise whole or partially fractionated postbiotic materials that are employed as biopreservatives, with the primary endpoint being the inhibition or control of foodborne pathogens or spoilage microorganisms within the food matrix rather than a demonstrated health benefit in the consumer. (iii) CFS and fermentates are compositionally complex products of microbial growth, consisting of spent culture media containing mixtures of organic acids, peptides, bacteriocins, and other microbial metabolites. These preparations represent the practical antimicrobial intervention evaluated in many cheese studies; however, they should be considered postbiotics in the sense of ISAPP only when a health benefit has been demonstrated. In the absence of such evidence, the terms “CFS” or “fermentate-based antimicrobial preparation” are more appropriate. (iv) Individual microbial metabolites, including purified bacteriocins, organic acids, or cyclic dipeptides, are chemically defined or purified antimicrobial compounds. Accordingly, an individual bacteriocin such as nisin should be classified as a bacteriocin rather than as a postbiotic, as referring to a single purified molecule as a postbiotic conflates a specific microbial metabolite with the broader concept of postbiotic preparation. Throughout this review, the term “postbiotic” is therefore reserved for preparations that are consistent with categories (i) and (ii), whereas “CFS” and “fermentate” are used for category (iii), and individual microbial metabolites are identified explicitly according to their specific nature (category iv). This distinction is intended to clarify that the antimicrobial evidence discussed below does not imply that all microbial-derived preparations automatically qualify as health-conferring postbiotics.

### 6.2. Evidence from Cheese and Dairy Matrices

The in-cheese evidence, though still limited, is now substantial enough to support real distinctions. The strongest controlled result remains the fermentate-versus-live-culture comparison of Engstrom and co-workers [133]: a cultured sugar-vinegar blend, a cell-free fermentate, at 1.0% completely inhibited *L. monocytogenes* for eight weeks in a refrigerated high-moisture model cheese and outperformed every commercial live protective culture tested in the same study. This represents the single most crucial finding for the field, as it isolates the metabolite fraction as the active driver of bioactivity and demonstrates that it outperforms the viable-cell comparator on its own terms. A postbiotic-fortified yogurt provides the clearest quantitative pathogen-reduction figure in a dairy matrix, cutting *L. monocytogenes* by roughly 2 log CFU over 21 days at 4 °C [134], and fruit-derived LAB drove down the same pathogen in Minas Frescal via organic-acid production [121]. The artisanal cheese environment is itself a productive reservoir of postbiotic-active strains: a survey of Brazilian artisanal cheeses found antagonistic activity in more than 94% of LAB isolates, with roughly an eighth producing bacteriocins and a similar fraction producing EPS [135], and CFS and BLIS preparations from cheese-isolated *Enterococcus*, *Pediococcus* and *Lactiplantibacillus* strains show reproducible anti-listerial and broad anti-pathogen activity [128,129,136]. Two features of this in-cheese literature deserve emphasis. Anti-biofilm and anti-virulence activity, not merely cell-count reduction, is a recurring and distinctive theme: postbiotic CFS preparations degrade pre-formed biofilms and suppress biofilm formation by *Salmonella* [137], MRSA [138], *S. aureus* from yeast-derived postbiotics [139], and several clinically relevant pathogens in cheese-isolate CFS [140,141]. This quorum-sensing and virulence-interference mechanism, reviewed by Che and co-workers [142], is a genuine point of differentiation from phages and from simple acid inhibition. Second, spoilage control, especially antifungal activity against *Aspergillus* and *Penicillium*, is at least as well evidenced as pathogen control, with cheese-applied CFS-plus-cyclic-dipeptide preparations extending shelf life against mold [143]. A methodological caution runs through all of it: Gajewska and colleagues [144] show that how the postbiotic is prepared (the inactivation and concentration method) materially changes the antibacterial activity recovered, so nominally identical preparations are not interchangeable.

### 6.3. Films, Coatings, and Whey Valorization

As with bacteriocins, the primary practical bottleneck for postbiotics in cheese is maintaining effective concentrations of the active fraction at contamination sites throughout a week-long ripening process. The prevailing strategy to address this limitation mirrors that of AMPs: integration into active edible films and coatings. A postbiotic-loaded chia-seed mucilage film raised the inhibition zone against *E. coli* O157:H7 in ultrafiltered cheese from 12.2 to 15.3 mm [145], and bacterial-nanocellulose films carrying CFS and cyclic dipeptides suppressed fungal growth on cheese surfaces [143]. A distinctively dairy-oriented approach involves whey valorization: several research groups produce postbiotics directly within cheese whey or ultrafiltered whey permeate and reapply them to cheese matrices. This strategy effectively converts a high-volume side-stream into a functional preservative feedstock [146,147]. Encapsulation represents an advanced technological solution to the same retention challenge: liposomal encapsulation of pediocin achieved ~89% encapsulation efficiency and sustained release while fully retaining antimicrobial activity [148]. Consequently, recent reviews increasingly highlight active edible packaging and freeze-dried postbiotic powders as the primary pathways toward achieving shelf-stable, dose-defined biopreservative formulations [126,127].

### 6.4. Postbiotics Versus Live Protective Cultures

Because live-culture biopreservation serves as the natural comparator, the underlying trade-off warrants explicit positioning. Live protective cultures boast a deeper, more consistent track record in cheese matrices and can achieve larger, sustained pathogen reductions; however, they introduce a viable organism with its own metabolic and regulatory ramifications. Conversely, postbiotics trade a degree of raw potency for superior kinetic stability, precise dose control, predictable matrix behavior, and an enhanced safety profile for vulnerable consumer demographics [126,149]. While the findings of Engstrom and colleagues [133] provide proof of concept that this compromise does not inevitably sacrifice antimicrobial efficacy, these data represent a single study. Across the broader literature, postbiotics consistently emerge as the more controllable, albeit generally less potent, biopreservation strategy.

### 6.5. Limitations

The limitations most specific to postbiotics stem from the compositional undefinedness of the preparations themselves. A CFS or fermentate is a variable mixture whose activity is difficult to standardize, a problem compounded by the finding that the inactivation–concentration method itself changes recovered activity [144]. Mechanistic attribution, though improving with metabolomics, remains incomplete for most preparations, so it is often unclear whether a given result would survive removal of the organic-acid fraction. The evidence base is also disproportionately review- and in vitro-heavy: many “postbiotic in cheese” papers characterize a CFS by agar diffusion or broth MIC and infer dairy applicability rather than running an in-cheese challenge trial to the end of shelf life. The regrowth problem documented for the whole biocontrol class [120], the incommensurable activity units, and the unresolved regulatory status of a non-viable fermentate are shared with the other modalities and are discussed comparatively in Section 7.

The available postbiotic preparations and cell-free metabolites assessed in cheese and related dairy applications are summarized in Table 3, together with their target microorganisms and the main preservation outcomes.

## 7. Conclusions

Bacteriophages, antimicrobial peptides, and postbiotics each provide viable strategies to enhance the microbiological safety of cheese while avoiding the sensory and nutritional degradation associated with thermal or chemical treatments. However, these modalities exist at distinctly different stages of technological readiness. Bacteriophages represent the most mature intervention, backed by regulation-cleared commercial formulations [61] and broad host ranges, yet their efficacy remains highly susceptible to the cold, acidic, and lipophilic environment of the cheese matrix. Antimicrobial peptides—anchored by the well-characterized benchmark nisin—benefit from a robust mechanistic foundation. Their primary bottleneck has shifted from agent discovery to localized delivery and retention of bioactivity in situ. Postbiotics represent the least mature, but most versatile approach, offering superior physical stability and favorable safety profiles for sensitive consumer demographics, albeit often at the expense of raw antimicrobial potency. Despite these operational differences, all three modalities converge on several core principles: combinatorial and hurdle strategies consistently outperform monotherapies, surface-targeted delivery serves as a universal engineering solution, and identical translational barriers—including target regrowth during extended ripening, matrix-induced binding or inactivation, non-standardized activity units, and ambiguous regulatory frameworks—hinder widespread adoption. Consequently, none of these biological strategies is currently positioned to replace conventional control measures independently, particularly given that the literature remains overly reliant on in vitro models and short-term challenge assays. Future advancement requires a shift in research priority from novel agent discovery toward rigorous translational validation. Specifically, the field demands full-shelf-life challenge trials in authentic cheese matrices using standardized dosages and harmonized storage conditions to evaluate these tools head to head alongside established controls. Rather than acting as standalone substitutes, biological interventions will find their highest utility integrated into multi-hurdle biopreservation systems that complement existing physicochemical, technological, and hygienic safeguards. Demonstrating efficacy under these standardized conditions is essential to transition these biological tools from promising laboratory concepts into validated components of industrial food.

Viewed comparatively, the three modalities are constrained by four cross-cutting limitations that recur irrespective of the agent, and their severity is stratified across the technology-readiness gradient. (1) Post-treatment regrowth over the full ripening period is the most consistently under-tested failure mode: it is documented for the whole biocontrol class [120], yet is rarely evaluated to the end of shelf life for any modality, and it is most acute for postbiotics, where long-duration in-cheese data are scarcest. (2) Matrix interference—binding to fat and casein and modulation by pH, salt, and calcium—systematically inflates in vitro estimates of in-cheese performance. It is most pronounced for phages (cold/acidic/fatty conditions) and AMPs (peptide binding), and comparatively less limiting for the more matrix-stable postbiotic metabolites. (3) Non-standardized activity units (PFU/g, AU/g, ppm, inhibition-zone diameters, log CFU, percent reduction) obstruct head-to-head comparison across all three fields and are compounded, for postbiotics, by the absence of any accepted potency standard. (4) Regulatory status is unresolved for each modality, but along different axes: phages sit between processing aid and additive with added scrutiny for engineered lysin strains, only nisin among AMPs holds clear GRAS/additive status, and the status of a compositionally undefined non-viable fermentate is entirely open.

A recurring, modality-spanning safety consideration concerns the biological agents and their producer strains, which is addressed here in consolidated form rather than dispersed across the preceding sections. For bacteriophages and their endolysins, the principal issues are the regulatory ambiguity of deliberately added lytic agents and the additional oversight required for genetically modified starter strains engineered to secrete lysins. For bacteriocins, the safety question is chiefly one of producer-strain suitability: enterococcal producers may carry transferable virulence and antibiotic-resistance determinants, so their use as live adjunct cultures demands strain-level screening even when bacteriocin potency is high, whereas purified or food-grade-produced peptides such as nisin avoid this concern. For postbiotics, the non-viability of the preparation removes the proliferation and producer-strain risks that attend live cultures, shifting the safety emphasis onto compositional characterization and reproducibility of the inactivated preparation. Explicit strain- and preparation-level safety assessment should therefore be reported alongside efficacy for all three modalities.

Beyond antimicrobial efficacy, industrial adoption will depend on economic and process-integration factors that the laboratory-scale literature has largely left unaddressed. The three modalities differ markedly on these criteria. Bacteriophage- and nisin-based preparations already exist as commercial products and can in principle be integrated into existing spraying, dipping, or packaging steps with limited retooling, although phage titer stability and batch-to-batch consistency during scaling up remain practical hurdles. AMP and encapsulated-peptide formats add cost and process steps (peptide production, encapsulation, active-film manufacture) that must be weighed against the shelf-life gain, while their in situ delivery via bacteriocinogenic starter or adjunct cultures is comparatively inexpensive but constrained by strain qualification. Postbiotics are potentially the cheapest to produce—particularly when generated from cheese or ultrafiltration whey as a valorization route—but the compositional variability of fermentates poses the greatest batch-to-batch standardization and quality-control challenge of the three. Across all modalities, realistic deployment will require costing studies, demonstration of compatibility with existing HACCP-controlled process flows, and evidence of stable performance across production batches at manufacturing scale.

Translating these tools into validated preventive measures requires a defined sequence of validation steps that the current literature only partially addresses. First, efficacy must be demonstrated in full-shelf-life challenge trials in the target cheese under realistic storage, using standardized inoculation levels and harmonized reporting units, and benchmarked against conventional controls, and for postbiotics against the live protective cultures they aim to replace. Second, sensory and physicochemical validation is essential, since an intervention that alters flavor, texture, or ripening dynamics is not adoptable regardless of its antimicrobial performance; formal sensory panels and consumer-acceptance testing are therefore an integral, and currently underreported, validation endpoint. Third, performance and stability must be confirmed at full production scale and across batches before an intervention can be written into a HACCP plan as a validated control measure at a critical control point or as a supporting prerequisite program. Incorporating “validation” explicitly as a research endpoint—and correspondingly as a search and indexing term—would help close the gap between proof-of-concept antimicrobial studies and implementation-ready interventions.

Although this review focuses on foodborne pathogens, the same biological interventions frequently exert useful secondary activity against spoilage microorganisms—for example, the antifungal action of dairy fermentates against Aspergillus and Penicillium and the anti-biofilm and biogenic-amine–limiting effects of certain bacteriocins—which can extend shelf life alongside safety benefits. Conversely, these strategies act on living targets and are not expected to mitigate chemical hazards such as drug residues or mycotoxins, which require control at the raw-milk and primary-production stage. This delimits the role of biocontrol within an integrated cheese-safety framework.

To highlight their different levels of technological maturity and practical applicability, Table 4 provides a comparative overview of bacteriophages, antimicrobial peptides, and postbiotics across the principal criteria relevant to cheese biocontrol.

## Figures and Tables

**Table 1:** Bacteriophages and endolysins.

Compound/Agent	Format/Type	Target Organism	Key Finding	Reference
ListShield (phage cocktail)	Commercial prep	*L. monocytogenes*	82% (0.7 log) reduction on cheese, no organoleptic change	[62]
PhageGuard/Listex P100 prep	Commercial prep	*L. monocytogenes*	~4 log reduction in pasteurized milk by day 7	[61]
Listex P100 in films/coated paper	Commercial prep + film	*L. monocytogenes*	Incorporated into biodegradable films; humidity critical to titer on paper	[63,64]
Phage treatment (condition study)	Lytic phage	*L. monocytogenes*	More effective at 22 °C than 6/14 °C, and at pH 6–6.5 than 5.5	[65]
Listex P100 + high-pressure processing	Lytic phage + hurdle	*L. monocytogenes*	Infective up to 300 MPa; phage + HPP dual-mechanism hurdle	[66]
Phage (calcium-modulated)	Lytic phage	*S. aureus*	Calcium concentration modulates adsorption and efficacy in cheese	[67]
EcoShield cocktail	Lytic phage cocktail	*E. coli* O157:H7	Up to 97% reduction at 5 × 10^6^–10^7^ PFU/g in cheese	[68]
Newly isolated lytic phage	Lytic phage	Enterotoxigenic *E. coli*	4.9–5.8 log CFU/mL reduction in cheese at 4 °C (largest in-cheese reduction)	[69]
Two-phage treatment	Lytic phage cocktail	*S. aureus*	Eradicated 10^6^ CFU/g in Cheddar curd after 14 d at 4 °C, no enterotoxin overproduction	[70]
phiIPLA-LAVI (myovirus)	Lytic phage	*S. aureus*	Kills in cheese matrices; stable across pH 4–7	[71]
Phage FA67	Lytic phage	*C. tyrobutyricum*	Delayed late-blowing defect by 2 weeks; reduced vegetative cells at 14 d ripening	[72]
Phage-loaded alginate films	Phage + film	*E. coli/P. fluorescens*	Cut biofilms by 1.7 and 2.7 log with biomass removal	[73]
PlyP100 endolysin	Endolysin	*L. monocytogenes*	Anti-listerial in fresh cheese ≥4 weeks; optimal at cheese pH and salt	[74,75]
LysFA67 endolysin	Endolysin	*C. tyrobutyricum*	Lysed 93% of target cells within 4 min	[76]
CTP1L endolysin (engineered L. lactis)	Engineered in situ starter	*Clostridium* spp.	Starter engineered to secrete lysin continuously in the matrix	[76,77]
Lysdb endolysin (engineered Lb. casei)	Engineered in situ starter	*S. aureus*	10^5^-fold reduction; held below 10^4^ CFU/g through 6 weeks ripening	[78]
GFP-fused CTP1L cell-binding domain	Endolysin (detection)	*Clostridium* spp.	Labels vegetative cells and spores with strain specificity	[79]
LysGH15 CHAP catalytic domain	Endolysin (catalytic domain)	*S. aureus*	Retains anti-staphylococcal activity in milk	[80]
Lysins + outer-membrane permeabilizers	Engineered endolysin	*Salmonella/E. coli*	Fusion reaches Gram-negative peptidoglycan	[81,82]

**Table 2:** Peptides and bacteriocins with antimicrobial effects.

Compound/Agent	Format/Type	Target Organism	Key Finding	Reference
GMp7 (milk-derived peptide)	Free AMP	*S. aureus*	Multi-target: peptidoglycan synthesis, ATP transport, and DNA replication	[91]
Novel enterocins (artisanal cheese)	Class-II bacteriocin	*L. monocytogenes*	Heat-stable to 80–100 °C, active across broad pH	[90]
Enterocin A-B-P—*E. faecium* KE82	In situ bacteriocinogenic starter	*L. monocytogenes*	0.54–2.00 log reduction during Galotyri ripening at 4–12 °C; in situ genes transcribed	[94,95]
Nisin-producing *Lactococcus lactis*	In situ starter	*L. monocytogenes/S. aureus*	In-fresh-cheese control of both pathogens	[97]
Purified in situ enterocin	Bacteriocin	*L. monocytogenes*	Undetectable at 2048 AU/g over 72 h	[92]
Nisin + lacticin 3147 + plantaricin starter	Engineered multi-bacteriocin starter	*L. monocytogenes*	Broadened spectrum; reduced single-bacteriocin resistance escape	[98]
Recombinant enterocin A producer	Engineered starter	*L. monocytogenes*	Multi-log reduction	[99]
Lactococcus lactis strain	Bacteriocinogenic starter	*L. monocytogenes*	3-log reduction over 10 d in goat cheese	[93]
Free nisin	Free peptide	*L. monocytogenes*	Inhibits in Queso fresco/processed cheese; pH-dependent MIC model at ~0.5–5 ppm	[100,101]
Staphylococcins	Free peptide	*S. aureus*	95.00–99.99% reduction in fresh Minas cheese	[102]
Nisin + caprylic acid + trans-cinnamaldehyde	Combination	*Salmonella*	Completely suppressed in queso fresco where nisin alone could not	[103]
Chitosan-coated nisin-silica liposomes	Encapsulation	*L. monocytogenes*	Sustained anti-listerial activity in Cheddar, no sensory penalty	[104]
Alginate/resistant-starch nisin microcapsules	Encapsulation	*C. tyrobutyricum*	Controlled nisin release at production scale	[105]
Neusilin/zein-starch nisin nanoparticles	Encapsulation	(extended release)	Nanoparticle carriers for extended release	[106]
Nisin-chitosan-cellulose film	Active film	*L. monocytogenes*	Complete inactivation on UF cheese; active 1 month at 4 °C	[107]
Enterocin whey-protein films	Active film	*L. monocytogenes*	Undetectable within 20–30 d; blocks migration between slices	[108]
Spray-dried enterocins (agar coating)	Coating	*L. monocytogenes*	1.0 log CFU/mL reduction in commercial goat cheese	[109]
Lactobacillus curvatus bacteriocin films	Active film	*L. monocytogenes*	Control on sliced Prato cheese	[110]
Nisin-loaded amaranth:pullulan nanofibers	Electrospun fiber	*L. monocytogenes*	Complete inactivation in fresh cheese within 120 h	[111]
PlyP100 endolysin + nisin	Combination (endolysin + bacteriocin)	*L. monocytogenes*	Non-enumerable in queso fresco over 4 weeks, no resistant escape (paradigm result)	[112]
Engineered nisin H27/31K + PlyP100	Combination	*L. monocytogenes*	Non-enumerable control in 14 days	[114]
Enterocin OS1 + eucalyptus essential oil	Combination (bacteriocin + EO)	*L. monocytogenes*	Undetectable in Moroccan fresh cheese within 2 days	[115]
Nisin A	Bacteriocin (quality application)	Biogenic-amine-producing LAB	Inhibited 31/48 BA-producers; prevented biofilm in two-thirds of formers	[118]
Staphylococcins/LAB bacteriocins	Free AMP	Drug-resistant *S. aureus*/*MRSA*	Control of MRSA from cheese isolates	[102,117]
Novel *Pediococcus*/*Enterococcus* bacteriocins	Bacteriocin	*L. monocytogenes*	Characterized against *Listeria* with quantified titers	[119]

**Table 3:** Postbiotics.

Compound/Agent	Format/Type	Target Organism	Key Finding	Reference
Fruit-derived *L. plantarum/L. paracasei* CFS	Cell-free supernatant (organic acids)	*L. monocytogenes*	Reduced in Minas Frescal, tracking lactic/acetic acid and pH drop	[121]
*L. plantarum* CFS (Koupa cheese isolate)	CFS (metabolomics-resolved)	*S. aureus*/*E. coli*	Activity attributed to nocardamine and diketopiperazines	[122]
Antifungal dairy fermentates	Fermentate	*Aspergillus*/*Penicillium*	10-OH-18:1 fatty acid + acetate explain >half of antifungal activity	[123,124]
LAB antifungal metabolites	CFS	Fungi	Activity correlated with defined organic acids and phenolics at measured MICs	[125]
BLIS/bacteriocin-containing CFS (cheese isolates)	CFS/BLIS	Broad (anti-listerial)	BLIS/bacteriocin CFS anti-listerial; heat-stable to 121 °C and active across a broad pH range	[128,129]
Cultured sugar-vinegar fermentate (1.0%)	Cell-free fermentate	*L. monocytogenes*	Complete inhibition 8 wk in model cheese; beat every live protective culture tested	[133]
Postbiotic-fortified yogurt	Postbiotic	*L. monocytogenes*	~2 log CFU reduction over 21 days at 4 °C	[134]
LAB from Brazilian artisanal cheeses	CFS (survey)	Broad	Antagonistic activity in >94% of isolates; ~1/8 bacteriocin, ~1/8 EPS producers	[135]
*Enterococcus*/*Pediococcus*/*Lactiplantibacillus* CFS-BLIS	CFS/BLIS	*L. monocytogenes* (broad)	Bacteriocinogenic *Enterococcus hirae* and *Pediococcus pentosaceus* strains from Brazilian artisanal cheese with anti-listerial activity [131], and *Lactiplantibacillus plantarum* strains from Slovak raw-sheep-milk cheese carrying plantaricin genes (plnEF, plnD, plnA, plnC) that inhibited six foodborne pathogens including *L. monocytogenes* by disc diffusion [136] (author to insert the specific anti-listerial titre, in AU/mL, reported by Cavicchioli et al. [131])	[128,136]
Postbiotic CFS	CFS (anti-biofilm)	*Salmonella*	*L. pentosus* YXJ1401 CFS degraded pre-formed biofilm and suppressed formation of multidrug-resistant Salmonella Enteritidis	[137]
Postbiotic CFS	CFS (anti-biofilm)	MRSA	Camel-milk *Lentilactobacillus parabuchneri* L-185 acid-associated metabolites showed anti-biofilm activity against MRSA	[138]
Yeast-derived postbiotic	Postbiotic	*S. aureus*	Indigenous cheese-derived yeast preparation showed anti-biofilm activity against *S. aureus*	[139]
Cheese-isolate CFS	CFS	Multiple clinical pathogens	Culture filtrates of *Lactiplantibacillus plantarum* (isolate L1) produced the largest inhibition zones on Congo red agar—32.33 mm against *S. aureus* and 35.33 mm against *E. coli*—within a panel also including *K. pneumoniae* and *P. aeruginosa*, with biofilm disruption confirmed by SEM/TEM and microtiter-plate assays [140]; *L. acidophilus* TMPC 41127 cell-free supernatant inhibited Salmonella Typhi biofilm formation dose-dependently, from 79.52% (¼× MIC) to 13.16% (4× MIC), and downregulated biofilm/invasion genes (csgD, csgA, bcsA, invA, invC) [141]	[140,141]
CFS + cyclic dipeptide (bacterial-nanocellulose film)	Postbiotic active film	*Aspergillus*/*Penicillium*	Suppressed fungal growth; extended cheese shelf life	[143]
Chia-seed mucilage postbiotic film	Active film	*E. coli O157:H7*	Inhibition zone rose from 12.2 to 15.3 mm on UF cheese	[145]
Whey-produced postbiotics	Fermentate (whey valorization)	(preservative feedstock)	Produced in cheese/UF whey and applied back to cheese	[146,147]
Liposome-encapsulated pediocin	Encapsulation	(controlled release)	~89% encapsulation efficiency, retained activity	[148]
Postbiotic preparation (method study)	CFS	(methodological)	Inactivation/concentration method materially changes recovered activity	[144]

**Table 4:** Comparative overview of the three biocontrol strategies for improving the microbiological safety of cheese.

Feature	Bacteriophages and Endolysins	AMPs & Bacteriocins	Postbiotics (CFS/Fermentates)
Main target organisms	*L. monocytogenes* (dominant); *Salmonella*, STEC/*E. coli*, wider dairy pathogens; endolysins extend Gram-positive reach	Gram-positive, above all *L. monocytogenes*; Gram-negatives intrinsically resistant unless combined with permeabilizers	Broad: *Listeria*, *Salmonella*, *S. aureus*/MRSA, *E. coli*, plus spoilage fungi (*Aspergillus*, *Penicillium*)
Spectrum of activity	Narrow and strain-specific (receptor-dependent); broadened by cocktails; endolysins broader within Gram-positives	Narrow to moderate; single peptides spectrum-limited; combinations widen it	Broad but non-specific; multi-metabolite mixtures acting via acids, bacteriocins, biosurfactants, anti-biofilm/anti-virulence effects
Major advantages	Multi-log reductions; self-replicating; no organoleptic change at effective doses; existing commercial products	Most extensively studied; well-characterized; nisin GRAS; potent against Listeria; combinable with endolysins	Non-viable (safe for vulnerable consumers); heat- and pH-stable; dose-controllable; anti-biofilm/anti-virulence action; whey-valorization route
Main limitations	Resistance (receptor-loss mutants); efficacy collapses in cold/acidic/fatty matrix; consumer “virus” perception; regrowth under-tested	Listeria develops tolerance; fat/casein binding lowers delivered dose; only nisin clearly authorized; enterococcal safety concerns	Review-/in vitro-heavy; no dose standardization (incommensurable units); variable composition; incomplete mechanistic attribution
Sensitivity to cheese matrix	High—performance strongly reduced by low temperature, low pH and fat (broth/milk results overstate efficacy)	High—fat, casein, salt and calcium bind peptide and cut the effective dose	Moderate—acid-based activity partly matrix-dependent; stable metabolites tolerate processing better
Delivery options	Direct spray/dip, milk addition, cocktails, hurdle combinations, lysin-secreting starters, active films	Direct addition, producer (adjunct) cultures, encapsulation, active/edible films and coatings	Direct application, active edible films/coatings, encapsulation (e.g., liposomes), whey-based fermentates, freeze-dried powders
Technological maturity	Highest—commercial products (Listex/PhageGuard P100, ListShield, EcoShield, SalmoFresh); endolysins emerging	High—nisin in industrial use; broad research base; delivery formats maturing	Lowest—mostly in vitro/model-cheese studies; few full-shelf-life trials
Regulatory readiness	Several GRAS/equivalent clearances; ambiguous processing-aid vs additive status; GMO issues for engineered lysins	Only nisin has GRAS/additive status; newer enterocins and engineered derivatives unauthorized	Unresolved—non-viable fermentate status (processing aid/additive/culture) undefined; compositional variability a barrier
Key research gaps	Full-shelf-life challenge trials in real cheese at defined titers; resistance/regrowth durability; harmonized reporting units	Long-duration trials capturing resistant regrowth; defined doses/formats; harmonized units; safety of non-nisin producers	Full-shelf-life in-cheese trials with defined, reproducible preparations; potency standardization; benchmarking vs. live protective cultures

## Data Availability

No new data were created or analyzed in this study. Data sharing is not applicable to this article.

## Abbreviations

The following abbreviations are used in this manuscript.

AMP	Antimicrobial peptide
ATP	Adenosine triphosphate
AU	Arbitrary units (of bacteriocin activity)
BLIS	Bacteriocin-like inhibitory substance(s)
CFS	Cell-free supernatant
CFU	Colony-forming unit(s)
CHAP	Cysteine, histidine-dependent amidohydrolase/peptidase (endolysin catalytic domain)
CIP	Cleaning in place
CPS	Coagulase-positive staphylococci
DNA	Deoxyribonucleic acid
EHEC	Enterohemorrhagic Escherichia coli
EPS	Exopolysaccharide(s)
EU	European Union
GFP	Green fluorescent protein
GMO	Genetically modified organism
GRAS	Generally recognized as safe
HPP	High-pressure processing
HUS	Hemolytic uremic syndrome
ISAPP	International Scientific Association for Probiotics and Prebiotics
LAB	Lactic acid bacteria
MIC	Minimum inhibitory concentration
MRSA	Methicillin-resistant *Staphylococcus aureus*
PFU	Plaque-forming unit(s)
RTE	Ready to eat
SE	Staphylococcal enterotoxin(s)
SEA–SEE	Staphylococcal enterotoxin types A to E
SFP	Staphylococcal food poisoning
STEC	Shiga toxin-producing *Escherichia coli*
Stx1/Stx2	Shiga toxin 1/Shiga toxin 2
VTEC	Verocytotoxin-producing *Escherichia coli*
